# Supplementary material for: Effects of the Implementation of an Intervention Based on Falls Education Programmes on an Older Adult Population Practising Pilates–A Pilot Study
Source: Int J Environ Res Public Health. 2023 Jan 10;20(2):1246. doi: 10.3390/ijerph20021246 (PMC9859372; doi:10.3390/ijerph20021246)
Supplement: Supplementary file 1 [file ijerph-20-01246-s001.zip › ijerph-2035300-supplementary.pdf]

SESSION 1

**OBJECTIVES**	- Know the main features and benefits of the JUA program.<br>- Correctly perform the placement ofthe belt, greeting and basic positions.<br>- Start practisingdisplacement, balance and coordination exercises.
**CONTENTS**	JUA basics, scrolling exercises, basic positions, balance and coordination.
**MATERIAL RESOURCES**	1 judo belt and 1mat per person, 1 speaker and music.

	ACTIVITY	GRAPHIC
**15 min**	1.- "**Presentation of the JUA**": individually, users are arranged in a circle.<br>• **Characteristics of the JUA**: the technician explains the characteristics of the JUA.<br>• **Belt**: belt placement (coordination work).<br>• **Greetings**: explanation, demonstration and realisation of the kneeling salute.<br>▪ **Important to** teach how to sit and get up.	
	2.- "**Changes** of direction" :individually, users are distributed by space and walk in any direction (sliding front of the foot). At the voice of "YA", users choose another path to keep walking.<br>**VARIANT 1**: change direction and mode of travel)<br>▪ **Important** to dodge the rest of the teammates.	
**30 min**	**Pilates mats 10 min.**	
	1.- "**Basic positions**": basic natural position.<br>**VARIANT 1**: natural position with left leg forward.<br>**VARIANT 2**: natural position with right leg forward.	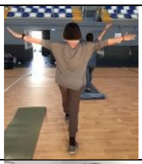
	2.- "**Displacement exercise**": individually, users imitate displacements made by the technician. Most displacements slide the front of the foot.<br>• **VARIANT 1**: imitate the movements of the person indicated by the technician.	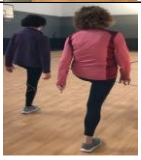
	3.- "**Balance exercise**": individually, users have to flex the right knee, lift the foot off the ground and bring the left elbow to the bent knee. Change leg and arm after 5 repetitions.<br>• **ADAPTATION**: whoever needs it can lean on the wall.	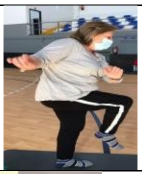
	4.- "**Balance exercise**": individually, users extend their right leg to the right side and tilt the trunk with their arms crossed to the opposite side. 3 times with each leg.<br>• **ADAPTATION**: whoever needs it can lean on the wall.	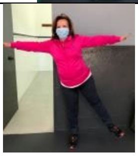
**15 min**	5.- "Coordination **exercise**": individually, users lie on the floor supinely, with arms extended towards the ceiling and legs flexed to 90 degrees.<br>They lower the right arm back and extend the left leg 5 times. After five repetitions they perform it with an opposite arm and leg.<br>**ADAPTATION**: whoever needs it, can be done sitting.	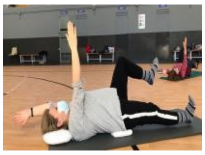
	1.- "**Stretching**": individually, users perform the stretches following the instructions of the technician.	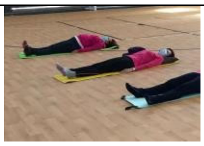

	2.- "**Conclusions** and **sharing**": individually users raise doubts, and the technician asks questions, such as:<br>What do you think of this first class of the JUA program?	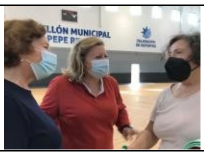

SESSION 2

**OBJECTIVES**	- Correctly perform the placement of the belt, greeting and basic positions.<br>- Start practicing displacement, balance and coordination exercises.
**CONTENTS**	Basic aspects of the JUA, displacement exercises, balance and coordination.
**MATERIAL RESOURCES**	1 judo belt and 1 mat per person, 1 speaker and music.

	ACTIVITY	GRAPHIC
**15 min**	1.- "**Initial routine**": individually, in each session users put on the belt and perform the greeting on their knees.<br>• **ADAPTATION**: perform standing if necessary.<br>• **ADAPTATION** : perform in a chair if necessary.	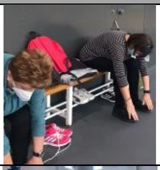
	2.- "**Touch back**": individually, users are spread out in the space and have to move by sliding the toes of the feet. In addition, they have to try to touch back the companions and prevent them from touching theirs.	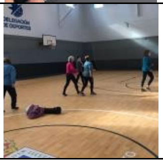
**30 min**	**Pilates mats 10 min.**	
	1.- "**Basic** positions ": individually remember the 3 positions of session 1.<br>**VARIANT 1**: they move through the space sliding feet and when the technician indicates one of the 3 positions, they have to perform it.	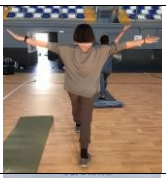
	2.- "Exercise of displacement, breathing and **postural control**": individually, the users perform a lateral displacement while raising their arms through a cross position. They breathe air through the nose and exhale through the mouth.	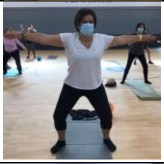
	3.- "**Balance exercise**": individually, users are placed with soles of their feet resting on the ground. They have to look for an imbalance by leaning on the toes of their feet.	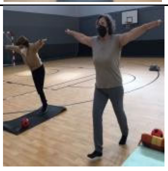
	4.- "**Balance exercise**": individually, users are placed with soles of their feet resting on the floor. They have to look for an imbalance by leaning on their heels.	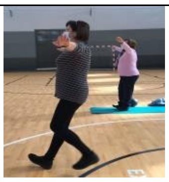
**15 min**	5.- "**Coordination exercise**": in pairs, they are placed facing each other. One partner raises their right or left arm, whilst the other person grabs their sleeve with the same arm. Swap roles.<br>**VARIANT 1**: grip with the opposite arm to the one raised by the partner.	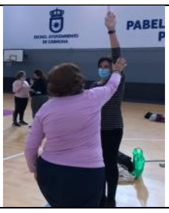

	1.- “**Stretching**": <u>individually,</u> users perform the stretches following the instructions of the technician.	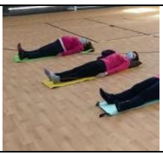
	2.- "**Conclusions** and **sharing**": <u>individually,</u> users raise doubts, and the technician asks questions, such as:<br>• Have you encountered difficulty in the exercises?What exercise do you think serves you best or can you apply in your daily life?	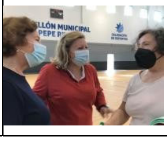

SESSION 3

**OBJECTIVES**	- Correctly perform the exercises of displacement and basic positions.<br>- Start practicing specific JUA exercises.<br>• Practice the basic grasping form.<br>• Work imbalances becoming aware of rebalancing strategies.
**CONTENTS**	Basic aspects of the JUA, displacement, balance and coordination.
**MATERIAL RESOURCES**	1 judo belt and 1 mat per person, folios with hands and feet, 15 cones (in this case we have used their footwear) and 1 speaker and music.

	ACTIVITY	GRAPHIC
**15 min**	1 .- "**Initial routine**": <u>individually</u>, in each session users put on the belt and perform the greeting on their knees.<br>• **ADAPTATION**: perform standing if necessary.<br>• **ADAPTATION**: perform in a chair if necessary.	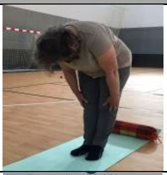
	2 .- "**Different forms of displacement**": <u>individually,</u> users move through the space, aiming to make large or small strides, as indicated by the technician, and always sliding feet across the front.<br>**VARIANT 1**: when the music stops look for a cone (shoe in this case).	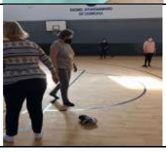
**30 min**	**Pilates mats 10 min.**	
	1.- "**Basic** positions ": <u>individually,</u> the users move and have to perform the positions according to the number indicated by the technician:<br>• **1**: Basic natural position.<br>• **2**: Natural position left leg forward.Natural position with right leg forward.	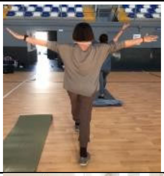
	2.- “Grip **exercise**": <u>in pairs</u>, they perform grips. One hand at elbow height and the other at shoulder height.<br>**VARIANT 1**: to the voice of "YA" they perform the learned grip.	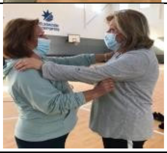
	▪ 3.- "**Imbalance exercise**": <u>in pairs</u>, they perform backward and forward imbalance exercises with the previous grip. Swap roles.	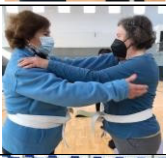
	4.- "**Range** of **motion exercise**": <u>individually</u>, users perform several mobility exercises following the instructions of the technician.<br>**Important**: focus on coxofemoral and glenohumeral joint.	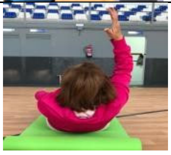

**15 min**	5.- “**Coordination exercise**": <u>individually,</u> users will have to "cross" from one side to another resting on the ground hands or feet taking into account the posters that they will find.	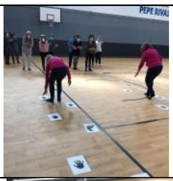
	• 1.- “**Stretching**": <u>individually,</u> users perform the stretches following the instructions of the technician.	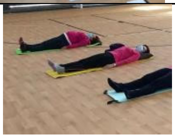
	2.- "**Conclusions** and **sharing**": <u>individually,</u> users raise doubtsand the technician asks questions, such as:<br>• How are you feeling as the sessions progress? Do these exercises give you security for tasks of your daily life?	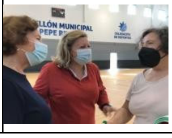

SESSION 4

**OBJECTIVES**	- Correctly perform the exercises of displacement and basic positions.<br>- Work on imbalances by becoming aware of rebalancing strategies.<br>- Control breathing.
**CONTENTS**	Exercises of displacements, grips, imbalances and breathing.
**MATERIAL RESOURCES**	1 judo belt and 1 mat per person, 1 speaker and music.

	ACTIVITY	GRAPHIC
**15 min**	1 .- "**Initial routine**": <u>individually</u>, in each session users put on the belt and perform the greeting on their knees.<br>• **ADAPTATION**: perform standing if necessary.<br>• **ADAPTATION**: perform in a chair if necessary.	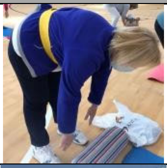
	2.- "**Cone slalom**": <u>individually,</u> users make movements from cone to cone (in this case we use weights). Every time they find a weight they have to touch it.<br>**VARIANT 1**: make the same route, but from behind.	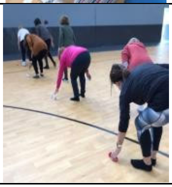
**30 min**	**Pilates mats 10 min.**	
	1.- "**Mobility** and breathing **exercises**": <u>individually,</u> users perform mobility **and breathing** exercises following the instructions of the technician.<br>• **ADAPTATION**: mobility exercises of the lower body that are carried out on the ground can be adapted. They perform them standing.<br>**Important**: emphasize breathing.	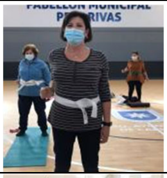
	2.- “Grip **exercise**": <u>in pairs</u>, they perform grips. One hand at elbow height and the other at shoulder height.<br>**VARIANT 1**: to the voice of "YA" they perform the learned grip.	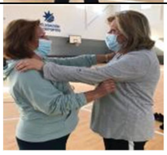
	3.- “**Imbalance** exercise": <u>in pairs</u>, perform imbalance exercises to the right and left with the grip of exercise 2. Swap roles.<br>**VARIANT 1**: imbalances forward, backward, right, and left.	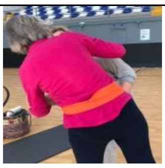

**15 min**	4.- "**Imbalance exercise**": <u>in pairs</u>, a member of the couple takes the belt and their partner grabs it at each end. The person who has removed the belt and grabs it by the part that are not the extremes, creates imbalances to their partner.	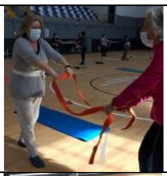
	• 1.- "**Stretching**": <u>individually,</u> users perform the stretches following the instructions of the technician.	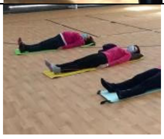
	2.- "**Conclusions** and **sharing**": <u>individually,</u> users raise doubts, and the technician asks questions, such as:<br>• Do you feel that you are "moving forward"?How are you feeling during the sessions?	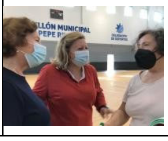

SESSION 5

**OBJECTIVES**	- Practice the basic grip form and imbalances.<br>- Improve rebalancing strategies.<br>- Start practicing specific JUA exercises.<br>• Make backward bearings with back to build confidence.<br>• Correctly execute simple backward fall exercises.
**CONTENTS**	Exercises of displacements, grips, imbalances and backward fall.
**MATERIAL RESOURCES**	1 judo belt and 1 mat per person, 1 speaker and music.

	**ACTIVITY**	**GRAPHIC**
**15 min**	1 .- "**Initial routine**": <u>individually</u>, in each session users put on the belt and perform the greeting on their knees.<br>• **ADAPTATION**: perform standing if necessary.<br>• **ADAPTATION** : perform in a chair if necessary.	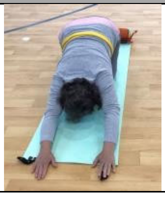
	2.- "**Joint** mobility ": <u>individually,</u> users perform joint mobility from bottom to top.	
**30 min**	**Pilates mats 10 min.**	
	1.- "**Displacement exercise**": <u>individually,</u> users have to perform different forms of displacement passing from one side of the rope to the other (we use the ground line).<br><u>Types of displacements</u>: normal sliding front of the foot, large strides, tiptoe, back.	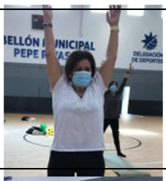
	2.- "**Imbalance exercise**": <u>In pairs</u>, perform imbalance exercises towards the right and left posterior corner with the learned grip. Swap roles.<br>**VARIANT 1**: imbalances forward, backward, right, left, rear right and left corner.	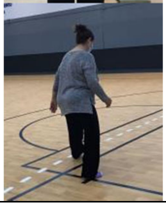

	3.- “**Bearing exercise**": <u>individually,</u> users perform a bearing with the back from a sitting position to lying down (lumbar, dorsal and cervical vertebrae).<br>• **ADAPTATION**: help yourself with your hands if necessary.<br>**VARIANT 1**: perform from lying down to sitting with bent knees.<br>**VARIANT 2**: with the knees extended (greater difficulty).	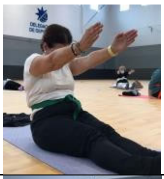
**15 min**	4.- “**Explanation of basic aspects of the fall back**": <u>individually,</u> users are arranged in a circle. The technician explains the basics of falls - protection of the neck and chin to the chest.<br><u>Individually</u>, people who did exercise number 3 without assistance can perform the fall backwards from the ground, taking into account the new indications.	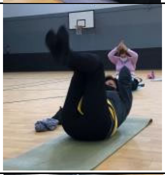
	• 1.- “**Stretching**": <u>individually,</u> users perform the stretches following the instructions of the technician.	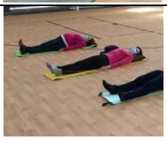
	2.- "**Conclusions** and **sharing**": <u>individually,</u> users raise doubts, and the technician asks questions, such as:<br>• Are you afraid, or do you have the feeling that you are going to fall in the exercises?Do you think that working on fall exercises can be useful for you?	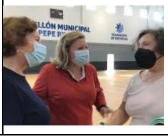

SESSION 6

**OBJECTIVES**	- Practice the basic grip form and imbalances.<br>- Improve rebalancing strategies.<br>- Make backward bearings with back to build confidence.<br>- Correctly execute simple backward fall exercises.
**CONTENTS**	Balance, imbalance and backward fall exercises.
**MATERIAL RESOURCES**	1 judo belt and 1 mat per person, 1speaker and music.

	**ACTIVITY**	**GRAPHIC**
**15 min**	1.- "**Initial routine**": <u>individually</u>, in each session users put on the belt and perform the greeting on their knees.<br>• **ADAPTATION**: perform standing if necessary.<br>• **ADAPTATION**: perform in a chair if necessary.	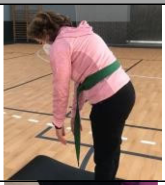
	2.- "**Joint** mobility": <u>individually,</u> users perform joint **mobility** from bottom to top, focusing on the joints involved in falls.	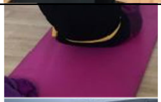
	**Pilates mats 10 min.**	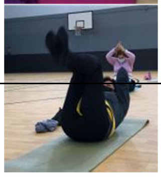
**30 min**	1.- “**Balance exercise**": <u>individually,</u> users "draw" a circle as wide as possible with the right and left foot, dragging on the ground.	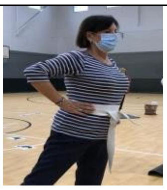

	2.- "**Imbalance exercise**": <u>in pairs</u>, perform imbalance exercises towards the right and left front corner. Swap roles.<br>**VARIANT 1**: imbalances forward, backward, right, left, rear right corner, left, front right and left corner.	
	3.- "**Bearing exercise**": <u>individually,</u> users perform bearings with their backs from a sitting position to lying down (lumbar, dorsal and cervical vertebrae).<br>• **ADAPTATION**: help yourself with your hands if necessary.<br>**VARIANT 1**: perform from lying down to sitting with bent knees.<br>▪ **VARIANT 2**: with the knees extended (greater difficulty).	
	4.- "**Balancing exercise**": <u>individually,</u> perform balancing exercises.<br>• **ADAPTATION**: help yourself with your hands if necessary.<br>▪ **Important**: take into account the basic aspects of the fall, explained in the previous session. Hands on the nape of the neck and chin on the chest.	
**15 min**	5.- "Backward fall **exercise**": <u>in pairs</u>, they grab each other's arms and a member of the couple performs the fall backwards from standing. Swap roles.<br>• **ADAPTATION**: If it is not possible to perform the exercise until sitting on the floor, users crouch as much as possible. Swap roles.<br>**Important**: previously perform leg strength exercises.	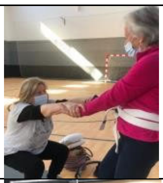
	• 1.- "**Stretching**": <u>individually,</u> users perform the stretches following the instructions of the technician.	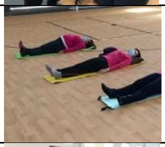
	2.- "**Conclusions** and **sharing**": <u>individually</u> users raise doubts, and the technician asks questions, such as:<br>Do you feel more confident as we go through the sessions?	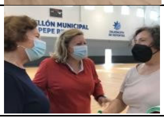

SESSION 7

**OBJECTIVES**	- Make backward bearings with back to build confidence.<br>- Correctly execute simple backward fall exercises.
**CONTENTS**	Fall back.
**MATERIAL RESOURCES**	1 judo belt and 1 mat per person, 2 balls, 1 speaker and music.

	**ACTIVITY**	**GRAPHIC**
**15 min**	1.- "**Initial routine**": <u>individually</u>, in each session users put on the belt and perform the greeting on their knees.<br>• **ADAPTATION**: perform standing if necessary.<br>• **ADAPTATION**: perform in a chair if necessary.	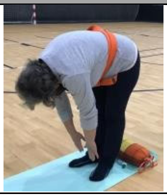
	2.- "**Joint** mobility": <u>individually,</u> users perform joint **mobility** from bottom to top, focusing on the joints involved in falls.	
	**Pilates mats 10 min.**	
	1.- "**Bearing set**": <u>individually,</u> the users are arranged in a circle, in the The technician is placed in the centre. A user throws the ball and makes a bearing.	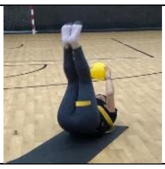

	2.- “Bearing **set**": <u>individually,</u> the users are arranged in a circle and with a ball they throw it between them and whoever receives it makes a bearing.<br>• **VARIANT 1**: include 2 balls.	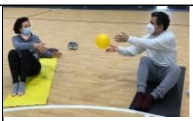
	3.- “Strength **exercises**": <u>individually,</u> users perform the strength exercises that the technician indicates.<br>• Right and left leg lifts with back attached to the wall.<br>• Squat.<br>• Stride.	
	4.- "Backward fall **exercise** ": <u>in pairs</u>, they grab each other's arms and a member of the couple performs the fall backwards from standing. Swap roles.<br>**ADAPTATION**: If it is not possible to perform the exercise until sitting on the floor, users crouch as much as possible. Swap roles.	
**15 min**	5.- “Fall **exercise**": <u>in pairs</u>, one person grabs the belt at the ends, their partner grabs it by the centre and performs from standing the fall back.	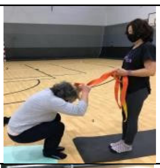
	• 1.- “**Stretching**": <u>individually</u> users perform the stretches following the instructions of the technician.	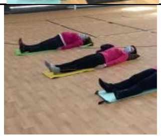
	2.- "**Conclusions** and **sharing**": <u>individually,</u> users raise doubts, and the technician asks questions, such as:<br>• Did you think you would be able to perform this type of exercise?Do you feel safe in the practice of these fall exercises?	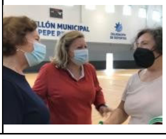

SESSION 8

**OBJECTIVES**	- Improve travel and coordination.<br>- Correctly execute the fall back.<br>- Start practicing specific JUA exercises.<br>• Work the lateral fall through simple exercises.
**CONTENTS**	Displacement, fall backward, fall sideways.
**MATERIAL RESOURCES**	1 judo belt, 1mat and 3 cones per person, speaker and music.

	ACTIVITY	GRAPHIC
**15 min**	1.- "**Initial routine**": <u>individually</u>, in each session users put on the belt and perform the greeting on their knees.<br>• **ADAPTATION**: perform standing if necessary.<br>• **ADAPTATION**: perform in a chair if necessary.	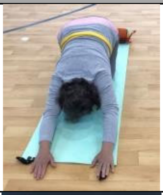
	2.-"**Colored cones**": <u>individually,</u> each user forms a triangle with their cones of different colours and is placed in the centre, when the technician indicates a colour the person moves and with the foot has to touch the cone of the corresponding colour.	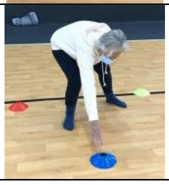
	**Pilates mats 10 min.**	

	1.- "**Strength exercises**": individually, users perform the strength exercises that the technician indicates.<br>• Right and left leg lifts with back attached to the wall.<br>• Squat.<br>• Stride.	
	2.- "**Backward fall exercise**": in pairs, review the two backward fall exercises from standing, with grip to the arms / hands of the partner and belt.<br>**ADAPTATION**: If it is not possible to perform the exercise until sitting on the floor, users crouch as much as possible. Change roles.	
**15 min**	3.-"Explanation of basic **sideways** fall": individually, all users are arranged in a circle and listen to the basic aspects of a lateral fall. They then perform a lateral fall from sitting.<br>**VARIANT 1**: Perform lateral fall from knees.	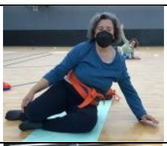
	• 1.-"**Stretching**": individually, users perform the stretches following the instructions of the technician.	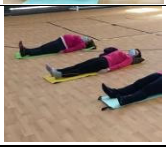
	2.- "**Conclusions** and **sharing**": individually, users raise doubts **and** the technician asks questions, such as:<br>• Did you think you would be able to perform this type of exercise? Do you feel safe in the practice of these fall exercises?	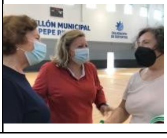

SESSION 9

**OBJECTIVES**	- Control breathing .<br>- Improve rebalancing strategies.<br>- Work the fall back and side.<br>- Perform reaction rate jobs.
**CONTENTS**	Backward fall, sideways fall, imbalances and reaction speed works.
**MATERIAL RESOURCES**	1judo belt and 1 mat per person, 10 tennis balls, 1 speaker and music.

	ACTIVITY	GRAPHIC
**15 min**	1.- "**Initial routine**": individually, in each session users put on the belt and perform the greeting on their knees.<br>• **ADAPTATION**: perform standing if necessary.<br>• **ADAPTATION**: perform in a chair if necessary.	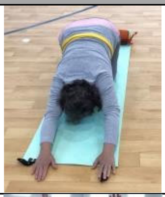
	2.- "**Joint** mobility": individually, users perform joint **mobility** from bottom to top, focusing on the joints involved in falls.	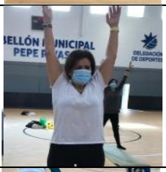
	**Pilates mats 10 min.**	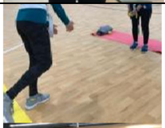
**30 min**	1.- "**Mobility** and breathing **exercises**": individually, users perform mobility and breathing exercises following the instructions of the technician.<br>• **ADAPTATION**: mobility exercises of the lower body that are carried out on the ground can be adapted. They perform them standing.<br>• **Important**: emphasize breathing	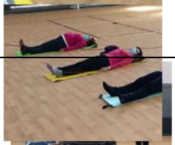 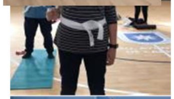 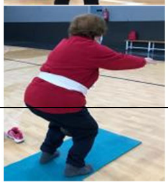

	2.- "**Strength exercises**": <u>individually,</u> users perform the strength exercises that the technician indicates.<br>• Right and left leg lifts with back attached to the wall.<br>• Squat.Stride.	
	3.- "**Backward fall exercise**": <u>in pairs,</u> review the two backward fall exercises from standing, with grip to the arms/hands of the partner and belt.<br>• **ADAPTATION**: If it is not possible to perform the exercise until sitting on the floor, users crouch as much as possible. Swap roles.<br>**VARIANT 1**: perform the exercise in scale.	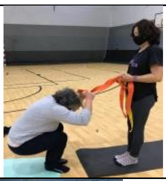
	4.- "**Lateral fall** exercise": <u>in pairs</u>, a member of the couple throws a ball and their partner has to catch it by performing lateral falls.	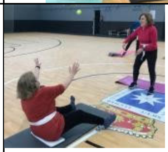
**15 min**	5.- "**Speed work**": in pairs, a member of the couple throws a tennis ball in different directions to create imbalances.	
	• 1.-"**Stretching**": <u>individually,</u> users perform the stretches following the instructions of the technician.	
	2.- "**Conclusions** and **sharing**": <u>individually,</u> users raise doubts, and the technician asks questions, such as:<br>• Do you like to work the contents in a playful way?How do you feel during the sessions?	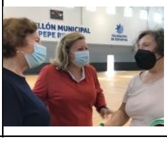

SESSION 10

**OBJECTIVES**	- Develop patterns of responses to different falls.<br>- Enhance functional stability.<br>- Standing again after a fall behind.<br>- Start practicing specific JUA exercises.<br>• Work hip technique to start the projection without falling to the ground.
**CONTENTS**	Displacement, fall back, lateral fall and hip projection technique.
**MATERIAL RESOURCES**	1judo belt and 1mat per person, 10 tennis balls, 1 speaker and music.

	ACTIVITY	GRAPHIC
**15 min**	1 .- "**Initial routine**": <u>individually</u>, in each session users put on the belt and perform the greeting on their knees.<br>• **ADAPTATION**: perform standing if necessary.<br>• **ADAPTATION**: perform in a chair if necessary.	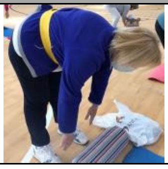
	2.- "**Joint** mobility": <u>individually,</u> users perform joint **mobility** from bottom to top, focusing on the joints involved in falls.	
	**Pilates mats 10 min.**	

	1.- "**Strength exercises**": individually, users perform the strength exercises that the technician indicates.<br>• Right and left leg lifts with back attached to the wall.<br>• Squat.Stride.	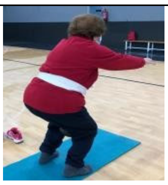
	2.- "Exercise of falls **back and side**": in pairs, users perform falls backwards and sides with grip to the arms/hands of the partner and belt and stand up.<br>• **ADAPTATION**: If it is not possible to perform the exercise until sitting on the floor, users crouch as much as possible. Swap roles.<br>**VARIANT 1**: if possible to perform without help.	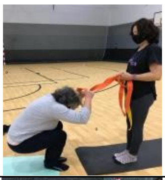
	3.- "**Tennis ball transport** exercise": individually, users sit on the floor. They take a tennis ball with their feet and move it from one side to the other.<br>• **VARIANT 1**: teams carry out a relay race.	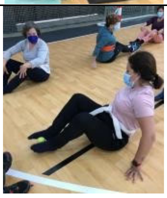
**15 min**	4.- "**Hip projection** technique": individually, users are arranged in a circle, listen to the basics of this technique and see a demonstration.<br>Next, they are placed in pairs and make the following progression:<br>• Gripping technique.<br>• Foot technique .<br>Projection technique.	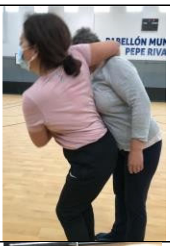
	• 1.-"**Stretching**": individually, users perform the stretches following the instructions of the technician.	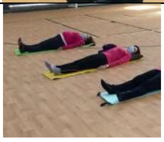
	2.- "**Conclusions** and **sharing**": individually, users raise doubts, and the technician asks questions, such as:<br>• Do you like how the sessions are planned?Do you find all the exercises useful?	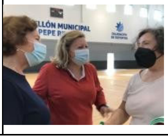

SESSION 11

**OBJECTIVES**	- Develop patterns of responses to different falls.<br>- Enhance functional stability.<br>- Work hip technique to start the projection without falling to the ground.
**CONTENTS**	Displacements, grip, imbalances, falls and projection techniques .
**MATERIAL RESOURCES**	1 judo belt, 1 mat and 1 bar per person, 1 speaker and music.

	ACTIVITY	GRAPHIC
**15 min**	1.- "**Initial routine**": individually, in each session users put on the belt and perform the greeting on their knees.<br>• **ADAPTATION**: perform standing if necessary.<br>• **ADAPTATION**: perform in a chair if necessary.	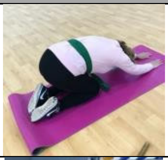
	2 .- "**Displacement exercise**": individually, execute displacements by the lines drawn on the ground.<br>**VARIANT 1**: game "the CoconutEater".	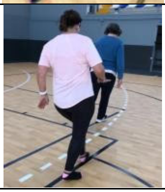

30 min	**Pilates mats 10 min.**	
	1.- "**Mobility** and breathing **exercises**": <u>individually,</u> users perform mobility and **breathing** exercises with a bar following the instructions of the technician.<br>• **ADAPTATION**: mobility exercises of the lower body that are carried out on the ground can be adapted. They perform them standing.<br>**Important**: emphasize breathing.	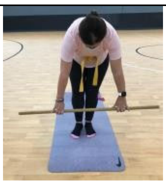
	2.-"**Imbalance exercise**": <u>in pairs</u>, perform imbalance exercises forward, backward, right, left, rear right corner, left, right and left front corner.<br>**VARIANT 1**: make the imbalance that the technician indicates out loud.	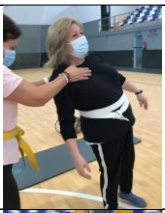
	3.- "Exercise of falls back and **side"**: <u>in pairs</u> users perform falls backwards and sides with grip to the arms/hands of the partner and belt.<br>• **ADAPTATION**: If it is not possible to perform the exercise until sitting on the floor, users crouch as much as possible. Swap roles.<br>• **VARIANT 1**: if possible to perform without help.	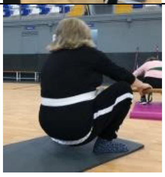
15 min	4.- "**Hip projection** technique": individually, users are arranged in a circle, remember the basics of this technique and see a demonstration.<br>Next, they are placed in pairs and make the following progression:<br>• Gripping technique.<br>• Foot technique .Projection technique .	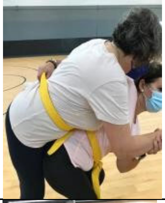
	1.- "**Stretching**": <u>individually,</u> users perform the stretches following the instructions of the technician.	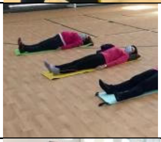
	2.- "**Conclusions** and **sharing**": <u>individually,</u> users raise doubts, and the technician asks questions.	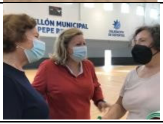

SESSION 12

**OBJECTIVES**	- Develop patterns of responses to different falls.<br>- Enhance functional stability .<br>- Review all the techniques, emphasising their application on a day-to-day basis.
**CONTENTS**	Displacements, grip, imbalances, falls, projection technique.
**MATERIAL RESOURCES**	1 judo belt, 1 mat and 1 bar per person, 1 speaker and music.

	ACTIVITY	GRAPHIC
15 min	1.- "**Initial routine**": <u>individually</u>, in each session users put on the belt and perform the greeting on their knees.<br>• **ADAPTATION**: perform standing if necessary.<br>• **ADAPTATION**: perform in a chair if necessary.	
	2.- "**Coloured cones**": <u>individually,</u> each user forms a triangle with their cones of different colours and is placed in the centre, when the technician indicates a colour the person moves and with the foot has to touch the cone of the corresponding colour.	
	**Pilates mats 10 min.**	

	1.- "**Mobility** and breathing **exercises**": individually, users perform mobility and **breathing** exercises with a bar, following the instructions of the technician.<br>• **ADAPTATION**: mobility exercises of the lower body that are carried out on the ground can be adapted. They perform them standing.<br>**Important**: emphasize breathing.	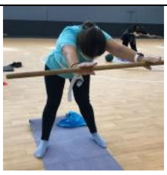
	2.-"**Imbalance exercise**": in pairs, perform imbalance exercises forward, backward, right, left, rear right corner, left , right and left front corner.<br>**VARIANT 1**: make the imbalance that the technician indicates out loud.	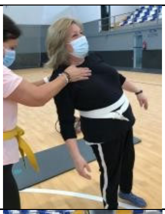
	3.- "Exercise of falls back and **side"**: in pairs, users perform falls backwards and sideways with grip to the arms/hands of the partner and belt.<br>• **ADAPTATION**: If it is not possible to perform the exercise until sitting on the floor, users crouch as much as possible. Swap roles.<br>• **VARIANT 1**: if possible to perform without help.	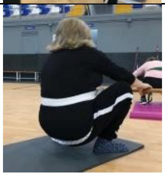
**15 min**	4.- "**Hip projection** technique": individually, users are arranged in a circle, remembering the basics of this technique and observing a demonstration.<br>Next, they are placed in pairs and make the following progression:<br>• Gripping technique.<br>• Foot technique .Projection technique.	
	1.-"**Stretching**": individually, users perform the stretches following the instructions of the technician.	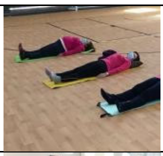
	2.- "**Conclusions** and **sharing**": individually, users raise doubts, and the technician asks questions.	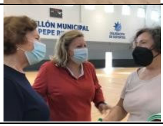
